# Exosomes as Emerging Non-Invasive Biomarkers of Cervical Cancer: A Systematic Review and Meta-Analysis

**DOI:** 10.3390/cancers17243945

**Published:** 2025-12-10

**Authors:** Fernanda Santos, Francisco A. Caramelo, Jorge M. P. Tomaz, Magda M. Santana, Rui J. Nobre, Luis P. Almeida, Margarida Figueiredo-Dias

**Affiliations:** 1 Faculty of Medicine, Gynaecology Department, University of Coimbra, 3004-504 Coimbra, Portugal; marg.fig.dias@gmail.com; 2 Coimbra Academic and Clinical Centre, 3000-075 Coimbra, Portugal; 3 Gynaecology Department, ULS—Coimbra Hospital and University Centre of Coimbra, 3004-561 Coimbra, Portugal; 4 Laboratory of Biostatistics and Medical Informatics (LBIM), Faculty of Medicine, University of Coimbra, 3000-548 Coimbra, Portugal; fcaramelo@fmed.uc.pt; 5 Centre for Innovative Biomedicine and Biotechnology (CIBB), University of Coimbra, 3004-504 Coimbra, Portugal; magdamsantana@gmail.com (M.M.S.); rui.jorge.nobre@gmail.com (R.J.N.); luispa@cnc.uc.pt (L.P.A.); 6 Blood and Transfusion Medicine Department at ULS Coimbra, ULS—Coimbra Hospital and University Centre of Coimbra, 3004-561 Coimbra, Portugal; jtomaz7@gmail.com; 7 Centre for Neuroscience and Cell Biology (CNC), University of Coimbra, 3004-504 Coimbra, Portugal; 8 Institute for Interdisciplinary Research (IIIUC), University of Coimbra, 3030-789 Coimbra, Portugal; 9 ViraVector, University of Coimbra, 3004-504 Coimbra, Portugal

**Keywords:** cervical cancer, extracellular vesicle, exosomes, cervical intraepithelial neoplasia, biomarkers, diagnosis, prognosis, miRNA, long non-coding RNAs, CtDNA

## Abstract

**Background/Objectives**: Cervical cancer remains a significant global health burden, underscoring the imperative for refined diagnostic and prognostic methodologies. This study aimed to evaluate the potential of extracellular vesicles (EVs) as non-invasive biomarkers for cervical cancer, focusing on diagnosis and prognosis. **Methods:** We conducted a systematic review and meta-analysis in accordance with PRISMA guidelines to assess the diagnostic and prognostic accuracy of EV-based biomarkers. We searched PubMed, EMBASE, and Web of Science for relevant studies. Twelve articles met the inclusion criteria: eight related to diagnostic accuracy, three to prognosis, and one to both outcomes. Six studies met the criteria for meta-analysis. We used a random-effects model to synthesise diagnostic data, while prognostic data were synthesised narratively. **Results:** The meta-analysis yielded a pooled area under the receiver operating characteristic curve (AUC) of 0.87 (95% CI 0.80–0.92) for EVs in the diagnosis of cervical cancer, indicating high accuracy. The evaluated diagnostic biomarkers were primarily non-coding RNAs. For prognosis, data heterogeneity precluded quantitative synthesis; however, individual studies identified diverse EV-associated molecules correlated with recurrence and survival. GRADE assessment indicated a high risk of bias and heterogeneity across studies. **Conclusions:** Extracellular vesicles demonstrate robust promise as diagnostic biomarkers for cervical cancer; however, their prognostic utility remains inconclusive due to methodological and clinical heterogeneity. Future research must prioritise the standardisation of isolation protocols and the execution of large-scale, prospective studies to validate EV biomarkers for clinical application. **Systematic Review Registration:** PROSPERO, identifier: CRD420251014411.

## Simple Summary

Cervical cancer persists as a significant global health burden, particularly in regions where screening and vaccination programmes are limited. Consequently, the identification of reliable, non-invasive biomarkers is paramount to enhance early detection and patient management. Extracellular vesicles (EVs) comprise lipid bilayer-enclosed particles released by cells into bodily fluids, carrying a molecular cargo that reflects the molecular signature of their cell of origin. This systematic review and meta-analysis assessed the diagnostic and prognostic utility of EVs in cervical cancer. Our findings indicate that EV-derived biomarkers, particularly non-coding RNAs, exhibit high diagnostic accuracy, yielding a pooled area under the receiver operating characteristic (ROC) curve of 0.87. However, evidence supporting their prognostic utility remains inconclusive due to methodological heterogeneity. In conclusion, EVs represent a promising liquid biopsy modality for cervical cancer detection; however, the validation of their clinical application necessitates further large-scale studies employing standardised protocols.

## 1. Introduction

Cervical cancer persists as the fourth leading cause of cancer-related deaths among women worldwide, underscoring its status as a significant public health concern [1]. Although incidence is relatively low in Western Europe, it continues to be the leading cause of cancer mortality among middle-aged women in Eastern European countries [1]. These global disparities are intrinsically linked to varying rates of Human Papillomavirus (HPV) infection, vaccination coverage, and the implementation of robust screening programmes [2]. Despite advancements in screening methods, including cytology and HPV testing, mortality rates have largely remained stable in developed countries [1,3]. Timely diagnosis is paramount, as early-stage detection allows for curative, fertility-sparing interventions and is associated with high survival rates. In contrast, advanced stages necessitate aggressive multimodal therapies and carry a significantly poorer prognosis [4]. Given that early-stage cervical cancer is often asymptomatic, elucidating disease progression through non-invasive means is crucial for timely diagnosis and monitoring [4]. Although histological examination remains the definitive diagnostic standard [4], conventional tissue biopsies may not accurately reflect the entire genomic and molecular landscape of a tumour due to intrinsic intratumoral heterogeneity [5]. As a non-invasive alternative, liquid biopsies offer a dynamic approach to cancer monitoring, providing real-time information on disease status and treatment response [5]. Circulating blood contains various tumour-derived materials, including circulating tumour cells (CTCs), circulating tumour DNA (ctDNA), and extracellular vesicles (EVs). Among these, EVs offer significant numerical and biological advantages as non-invasive biomarkers [6]

Extracellular vesicles (EVs), as defined by the International Society for Extracellular Vesicles (ISEV), encompass a heterogeneous population of lipid bilayer-enclosed particles released from cells [7]. EVs are broadly categorised by size and biogenesis: exosomes (typically 30–150 nm) are derived from the endosomal system and released upon the fusion of multivesicular bodies (MVBs) with the plasma membrane. In contrast, microvesicles (or ectosomes, generally 50 nm–1 μm) are generated by the direct outward budding and fission of the plasma membrane, while apoptotic bodies (1–5 μm) are larger fragments released during programmed cell death [7]. EVs are ubiquitous in most bodily fluids, and their concentration (often exceeding 10^9^ exosomes/mL) is significantly higher than that of circulating tumour cells (CTCs, <100 cells/mL) [6,8].

In the context of cancer diagnostics, exosomes have garnered particular attention due to their unique biogenesis and function as highly specific mediators of intercellular communication [9]. In contrast to microvesicles, which are often shed non-specifically via plasma membrane budding, or apoptotic bodies, which represent passive cellular remnants of programmed death, exosomes are actively secreted via the endosomal pathway [10]. This mechanism enables exosomes to carry a selective molecular cargo that accurately reflects the molecular status of the parental tumour cell [10]. Furthermore, the lipid bilayer structure of exosomes imparts inherent stability, actively protecting their enclosed cargo (including proteins and nucleic acids) from enzymatic degradation in circulation, a critical feature for effective biomarker analysis and validation [8].

Crucially, in cervical cancer, tumour-derived exosomes are not merely diagnostic markers but active mediators of oncogenesis [10]. They propagate the effects of HPV E6 and E7 oncoproteins by transferring specific viral transcripts, proteins, and non-coding RNAs to surrounding cells, thereby facilitating key tumour progression processes such as immune evasion, angiogenesis, and metastasis within the tumour microenvironment [11,12]. This capacity to encapsulate stable, functional, and disease-specific molecules directly involved in the pathogenesis of HPV-driven cancer distinguishes exosomes as the most relevant and studied EV subtype for liquid biopsy applications [11,12].

Extracellular vesicles can be isolated from various biofluids, most commonly plasma or serum, although urine, saliva, and cervicovaginal secretions have also been explored in gynaecological malignancies [7]. Their detection and analysis rely on complementary methodologies including ultracentrifugation, size-exclusion chromatography, nanoparticle tracking analysis, and transmission electron microscopy, ensuring both structural and molecular validation of vesicle integrity [7].

Consequently, this systematic review and meta-analysis aim to evaluate the potential of extracellular vesicles, with a specific emphasis on exosomes, as non-invasive biomarkers for the early diagnosis and prognosis of cervical cancer.

## 2. Materials and Methods

### 2.1. Protocol and Registration

This systematic review adhered to the Preferred Reporting Items for Systematic Reviews and Meta-Analyses (PRISMA) guidelines [13], the Grading of Recommendations, Assessment, Development and Evaluations (GRADE) approach for quality of evidence assessment [14], and the Quality Assessment of Diagnostic Accuracy Studies-2 (QUADAS-2) tool for the evaluation of risk of bias in individual studies [15]. The study protocol was registered prospectively in PROSPERO (registration number: CRD420251014411) prior to the commencement of the literature search.

### 2.2. Eligibility Criteria

Studies were eligible if they comprised original research or replication studies published in peer-reviewed journals, with full-text availability in English, Portuguese, or Spanish, without restriction regarding the year of publication.

In accordance with the PECO framework (Population, Exposure, Comparator, Outcome), specific inclusion and exclusion criteria are detailed in Table 1. Included observational studies were required to evaluate EVs as potential biomarkers and report specific quantitative data on EVs for cervical cancer detection or prognosis. Due to the limited number of studies anticipated in this emerging field, no restrictions regarding sample size were applied. Studies failing to report adequate details regarding EV isolation and characterisation methods were excluded, as were those focusing solely on therapeutic applications of extracellular vesicles without addressing their diagnostic or prognostic utility.

### 2.3. Information Sources

An initial scoping search was conducted in January 2025 to inform the development of the review protocol. This preliminary search facilitated the refinement of the research question and search strategy. Following the approval of the protocol by PROSPERO on 26 March 2025, a comprehensive systematic search was executed according to the pre-registered methodology. The search results were exported to EndNote 21.5 for Mac (Build 20846, Clarivate Analytics).

The following electronic databases were searched: PubMed, EMBASE, Cochrane Central Register of Controlled Trials (CENTRAL), Web of Science Core Collection, WHO International Clinical Trials Registry Platform, EU Clinical Trials Register, and ClinicalTrials.gov. Databases were searched from their inception until 30 April 2025 to ensure comprehensive coverage of this novel topic.

The search strategy utilised Boolean operators targeting title, abstract, and keywords fields with the following terms: (“cervical cancer” OR “cervical neoplasms” OR “uterine cervix cancer” OR “HPV-related cervical cancer”) AND (“Extracellular vesicl*” OR “plasma Extracellular vesicl*” OR “serum Extracellular vesicl*” OR “blood Extracellular vesicl*” OR “exosome” OR “microvesicles” OR “ectosomes” OR “oncosomes” OR “vesicular cargo” OR “liquid biopsy”) AND (“healthy” OR “cervical intraepithelial neoplasia” OR “CIN”) AND (“biomarker$” OR “biological biomarker” OR “diagnosis” OR “prognosis” OR “miRNA” OR “HPV” OR “ctDNA” OR “long non-coding RNAs (lncRNAs)”).

Additionally, annual conference abstracts from the International Papillomavirus Conference (IPVC) and the International Multidisciplinary HPV Congress (EUROGIN) from 2020 to 2025 were screened. Reference lists of included articles were manually scrutinised to retrieve potentially relevant studies that had not been identified in the initial search.

### 2.4. Study Selection

The screening process commenced on 4 January 2025. All duplicates were identified and removed automatically using EndNote 21.5. Two reviewers (F.S. and M.F.-D.) screened titles and abstracts independently and in a blinded manner, adhering to a predefined list of selection criteria. In instances where publications used overlapping patient cohorts, data were extracted from the study providing the most detailed information on EV biomarkers. A step-by-step comparison of the entire process was conducted, and any disagreements prompted a re-evaluation of the records in question. Eligibility was discussed among all authors involved in the selection process until consensus was achieved. The same procedure was applied to the scrutiny of reference lists of included studies to identify any additional relevant records.

From the selected articles, six were included for meta-analysis based on the availability of the Area Under the Curve (AUC). All studies assessed diagnostic outcomes. Both significant and non-significant results were considered for the analysis.

### 2.5. Data Extraction

All data variables to be extracted were defined a priori. A standardised data extraction spreadsheet was developed to minimize bias. The extracted variables were: (i) study, author, and publication year; (ii) country; (iii) study design; (iv) type of sample (e.g., plasma or serum); (v) EV subtype (e.g., exosome, microvesicle); (vi) EV isolation method; (vii) EV characterisation method; (viii) EV quantification method; (ix) specific biomarker identified (e.g., miRNA, lncRNA); (x) expression level (e.g., upregulated or downregulated); (xi) techniques used to identify and quantify the biomarkers; (xii) sample size (cervical cancer cases, healthy controls, and/or CIN controls); and (xiii) outcomes (e.g., diagnostic or prognostic) including descriptive statistics and association measures where available.

F.S. and M.F.-D. completed the initial data extraction independently. RN and MS subsequently reviewed the entire process, and any discrepancies were resolved by consensus. The data required for meta-analysis were collated by F.S. and M.F.-D. and subsequently reviewed by FC. JT and LPA provided a final verification of the quality of data entered into the database.

### 2.6. Risk of Bias in Individual Studies

The risk of bias in individual studies was evaluated using the Quality Assessment of Diagnostic Accuracy Studies-2 (QUADAS-2) tool [15], which assesses four key domains: patient selection, index test (in this case, extracellular vesicles as biomarkers), reference standard, and flow and timing.

Two independent reviewers (F.S. and M.F.-D.) performed the quality assessment for each included study. Each domain was assessed for risk of bias (high, low, or unclear) and applicability concerns. Following the initial assessment, results were compared, disagreements discussed, and a subsequent re-analysis performed. Finally, RN and MS reviewed the assessments to ensure the robustness of the evaluations.

The methodological quality of included studies was appraised considering key aspects of study design, conduct, analysis, and evaluation, informed by Simon et al.’s criteria [16] and the REMARK guidelines [17], whilst ensuring adherence to MISEV guidelines for EV research [7].

### 2.7. Summary Measures

Summary measures synthesised the diagnostic accuracy of exosomes as blood-based biomarkers.

For studies reporting multiple area under the ROC curve (AUC) values due to different experimental conditions, the lowest value corresponding to the worst-case scenario was selected, thus adopting a conservative approach. In one study, where the AUC was not directly available, it was estimated from the reported sensitivity and specificity values. For studies including an external validation group, AUC values obtained from these groups were prioritized.

Data were analysed using R (v 4.4.2) software [18]. A logit transformation was applied to AUC values to approximate a normal distribution, and the variance of the logit transformation was estimated for each study. A random-effects model was applied using the restricted maximum likelihood (REML) method through the ‘rma’ function of the ‘metafor’ package, accounting for the expected heterogeneity between studies. The summary measure and its 95% confidence intervals were calculated and subsequently back-transformed to the original AUC scale to facilitate interpretation.

Heterogeneity among studies was assessed using the Cochran Q test and the *I*^2^ statistic. The Q test evaluates whether observed differences in results are compatible with chance alone, with a *p*-value < 0.10 indicating significant heterogeneity. The *I*^2^ statistic quantifies the percentage of total variation across studies due to heterogeneity rather than chance, with values of 25%, 50%, and 75% suggesting low, moderate, and high heterogeneity, respectively [19,20]. The presence of publication bias was assessed visually through a funnel plot and quantitatively using Egger’s test. A *p*-value < 0.05 in Egger’s test indicated possible publication bias.

### 2.8. Confidence in Cumulative Evidence

Confidence in the cumulative evidence was assessed using the GRADE framework [14], adapted for observational studies. Observational studies start with a low-certainty rating, which can be upgraded or downgraded based on five dimensions: risk of bias (using tools such as QUADAS-2) [15], inconsistency (heterogeneity quantified by *I^2^* and Cochran’s Q) [20], indirectness (relevance of study populations and outcomes), imprecision (evaluated by 95% confidence intervals), and publication bias (assessed through funnel plots and Egger’s test). Sensitivity analyses were performed to exclude poor-quality studies. These assessments were performed independently by F.S. and M.F.-D. and verified by FC.

## 3. Results

### 3.1. Study Selection

**Figure 1:**
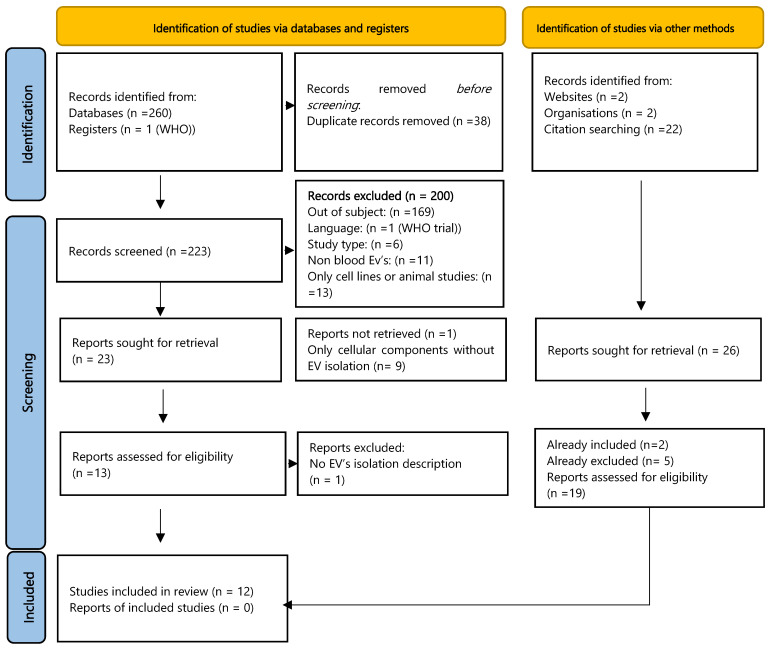
Flowchart describing the study selection process [13].

The electronic search across four databases yielded 260 records, with one additional record identified from clinical trial registry platforms. Following the removal of duplicates via EndNote (*n* = 27) and manual screening (*n* = 11), 223 unique records remained. After screening titles and abstracts, 23 studies were retained for full-text assessment. Subsequently, 12 studies met the eligibility criteria and were included in the qualitative analysis. (Figure 1).

Searches of websites and organisational resources identified four potential studies, and manual citation searching of included articles revealed 22 potentially relevant titles (including updated searches). Of these, two had already been included in our final sample, and five had been excluded during the process. Nineteen studies had not appeared in our initial searches; screening of their abstracts resulted in the exclusion of nine based on study type (e.g., abstract, review) and 10 based on failure to meet PECO criteria.

A meta-analysis was conducted on six articles related to cervical cancer diagnosis, focusing solely on extracellular vesicles as a collective biomarker entity, considering them as mediators or carriers of intercellular communication. Crucially, the specific biomarkers transported by isolated extracellular vesicles were not analysed individually due to their substantial heterogeneity across the included studies.

Among the included studies, the primary focus was on long non-coding RNAs (lncRNAs) [21,22], mRNA [23], plasma tRFs [24], and various miRNAs [23,25,26,27] or proteins [28,29]. Regarding prognosis, performing a meta-analysis was not feasible; although two studies reported AUC values [22,30], methodological disparities precluded the statistical pooling of results.

### 3.2. Study Characteristics

For the diagnostic evaluation, nine studies were selected, comprising a total of 659 cervical cancer patients (635 included in the meta-analysis), 482 healthy controls (458 included in the meta-analysis), and 139 CIN controls (all included in the meta-analysis) (Table 2).

For the prognostic evaluation, four studies were included (one of which assessed both outcomes), comprising 234 cervical cancer patients and 77 healthy controls (Table 3).

### 3.3. Results of Syntheses for Diagnosis

**Figure 2:**
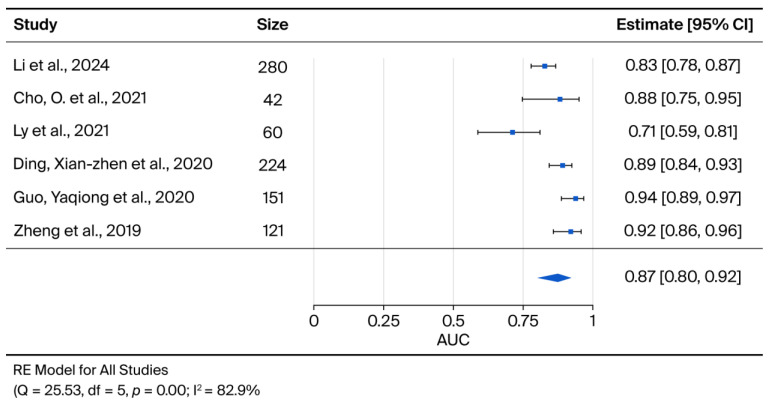
The forest-plot displays the Area under the curve (AUC) for each selected paper, along with the summary measure and its 95% confidence intervals.

The meta-analysis of the six studies meeting the eligibility criteria yielded a pooled AUC of 0.87 (95% CI: 0.80, 0.92), indicating robust diagnostic accuracy for exosomes as potential biomarkers for cervical cancer (Figure 2).

The age of participants varied considerably across the included studies, spanning a broad spectrum from <45 years [22] to >60 years [24,27] (details in Table 4). Reporting of Human Papillomavirus (HPV) status was inconsistent; while some studies documented HPV infection in a subset of cases (Positive: 97, Negative: 17) [21], others lacked this information [22,23]. Reported disease stages according to the FIGO classification ranged from early (I–II) to advanced (III–IV) disease.

Regarding exosome-specific biomarkers, differential expression patterns were observed in cervical cancer patients compared with healthy controls and/or individuals with CIN across various cargo types, including microRNAs (miRNAs) [23,25,26,27,30,31], lncRNAs [21,22], tRNA fragments (tRFs) [24], and proteins [28,29,32]. These findings suggest that these exosomal constituents mediate critical roles in tumorigenesis, immune modulation, and intercellular communication.

Several miRNAs, including miR-142-3p [23], miR-125a-5p [25], and a panel of eight miRNAs (let-7a-3p, let-7d-3p, miR-30d-5p, miR-144-5p, miR-182-5p, miR-183-5p, miR-215-5p, and miR-4443) [27], have been implicated in cervical carcinogenesis, potentially acting as oncogenes or tumour suppressors. Their exosomal enrichment and differential expression may influence signalling pathways governing proliferation, apoptosis, and metastasis.

Furthermore, the upregulation of lncRNA DLX6-AS1 [21] and lncRNA-EXOC7 [22] identified these transcripts as potential diagnostic biomarkers. The individual study AUCs for these specific biomarkers ranged from 0.71 for miR-125a-5p [25] to 0.94 for lncRNA-EXOC7 (analysed in serum and exosomes) [22]. Among the evaluated targets, lncRNA-EXOC7 appeared to possess the greatest potential for early diagnosis, demonstrating AUC values of 0.9388 and 0.8982 in serum and plasma, respectively [22] (Table 2).

Due to substantial variations in patient populations, subgroup analyses based on HPV status, histology, or disease stage were not feasible. Furthermore, statistical heterogeneity across the included studies was substantial (*I*^2^ = 82.9%), justifying the use of a random-effects model in the meta-analysis.

### 3.4. Results of Syntheses for Prognosis

Significant heterogeneity was observed regarding study populations and methodologies across the four studies included for prognostic analysis. Patient cohorts encompassed a broad age range, extending from early adulthood to the post-menopausal period. Histological subtypes were predominantly squamous cell carcinoma; however, several studies also included adenocarcinoma [30,31] and, less frequently, adenosquamous carcinoma [30,31]. Disease staging varied considerably, ranging from early (FIGO I–II) to advanced stages (FIGO III–IV) (Table 3 and Table 5).

Regarding biomarkers, considerable methodological variability was evident, particularly concerning the isolation and quantification of exosomes. Furthermore, the lack of consensus regarding reference values for exosome concentrations in healthy versus diseased individuals limited the interpretability of findings.

Among the included studies, the analysis by Someya et al. [30] demonstrated that a nine-miRNA signature (miR-148a-5p, 1915-3p, 3960, 183-5p, 196b-5p, 200c-3p, 182-5p, 374a-5p, and 431-5p) held the most significant prognostic potential, exhibiting differential expression between patients with no evidence of recurrence and those experiencing relapse. Further analysis revealed an inverse correlation between miR-374a-5p/miR-431-5p and tumour-infiltrating T cells (CD8+ and FOXP3+), implying a possible role in immune suppression and resistance to concurrent chemoradiotherapy (CCRT), thus underscoring its potential in personalised cervical cancer management [30].

### 3.5. Reporting Biases

**Figure 3:**
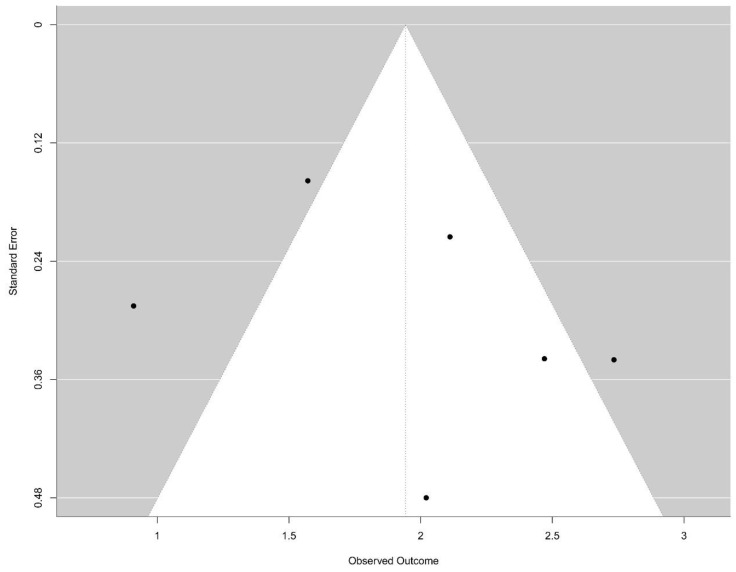
Funnel-plot: There is no evidence of bias publication (Egger test, *p* = 0.436).

Potential publication bias related to diagnostic outcomes was assessed using Egger’s test, yielding a *p*-value of 0.436, which suggests no significant evidence of publication bias (Figure 3).

Visual inspection of the funnel plot revealed a relatively symmetrical distribution of studies around the pooled effect size; however, the limited number of included studies (*n* = 6) restricted the statistical power to detect true asymmetry. Regarding prognostic outcomes, the included studies exhibited several limitations and high heterogeneity in study design and reporting quality.

The GRADE assessment indicated that confidence in the pooled estimates from this review is significantly compromised, primarily due to a high risk of bias within individual studies, marked inconsistency across findings, potential indirectness stemming from sample and methodological heterogeneity, and evidence of imprecision. Collectively, these factors preclude firm conclusions regarding the investigated diagnostic modalities (Table 6). A summary of the risk of bias identified across studies is detailed in Table 7.

## 4. Discussion

### 4.1. Summary of Main Results

This systematic review and meta-analysis reinforce the emerging paradigm of extracellular vesicles, specifically exosomes, as promising non-invasive biomarkers, particularly in the context of cervical cancer. The high diagnostic accuracy identified, with a combined AUC of 0.87 (95% CI: 0.80, 0.92), underscores their potential for early detection. The study also highlighted that the prognostic utility of extracellular vesicles remains inconclusive, a finding constrained by significant methodological heterogeneity among the included studies.

### 4.2. Results in the Context of Published Literature

The high diagnostic accuracy found in our review is consistent with results from studies in other tumour types, such as gastric [33], colorectal [34], and breast cancer [35], where EV-associated biomarkers have demonstrated AUC values ranging from 0.81 to 0.91.

These findings underscore the potential biological advantages of liquid biopsies over conventional methods, particularly regarding the quantitative characteristics of these analytes. Mechanistically, this diagnostic sensitivity is underpinned by the high abundance of circulating extracellular vesicles. In contrast to rare circulating tumour cells (<10 cells/mL), exosomes are present in plasma at concentrations typically ranging from 10^9^ to 10^12^ particles/mL, providing a vast reservoir of tumour-derived material for downstream analysis [36]. Furthermore, while absolute diagnostic cutoff values vary across studies due to differing normalisation strategies, the discriminatory power of these biomarkers is driven by the magnitude of their differential expression. For instance, our analysis highlighted markers such as lncRNA-EXOC7, which exhibited pronounced fold-changes, ranging from 2.93- to 3.18-fold elevations in cervical cancer patients relative to healthy controls. Such substantial signal-to-noise ratios are critical for early detection, enabling the identification of disease prior to the onset of symptoms [22].

These results emphasise the broad potential of extracellular vesicles as non-invasive diagnostic tools across various malignancies, which is crucial given the limitations of conventional diagnostic methods and the often advanced stage at diagnosis [37].

Consistent with other cancers, cervical cancer exhibits differential expression patterns of EV-associated molecules, including miRNAs, lncRNAs, and proteins. This suggests shared mechanisms in cancer progression and immune modulation across various malignancies. Moreover, the discovery of specific biomarkers, like lncRNA-EXOC7, with high diagnostic potential echoes findings for other tumours, where unique EV cargoes correlate with distinct cancer types [37].

However, a recurring theme in the broader EV literature, and a pivotal finding in our analysis, is the considerable heterogeneity among studies. This methodological variability is not unique to cervical cancer research and is a primary reason why our review was unable to perform a meta-analysis on the prognostic utility of extracellular vesicles. This limitation is also evident in the literature on endometrial cancer, where EV-associated proteins have shown high diagnostic accuracy and links to recurrence risk [38], yet studies on EV-derived miRNA signatures have revealed substantial inconsistencies, with only a minority of differentially expressed miRNAs overlapping between vesicular and whole serum analyses [39]. Similarly, while EV-associated miRNAs such as miR-21-5p and miR-214-3p have been implicated in predicting chemoresistance in ovarian cancer [40], clinical studies remain limited by small sample sizes and considerable variability in detection platforms [41,42]. In breast cancer, meta-analyses have identified EV-associated proteins like PD-L2 and sHLA-G as robust prognostic markers for overall and disease-free survival [43]. Furthermore, the presence of PD-L1-positive extracellular vesicles has shown potential in predicting response to immunotherapy, particularly in triple-negative breast cancer subtypes [44]. Nevertheless, conflicting associations with patient outcomes persist, mainly due to tumour heterogeneity and divergent EV detection and characterisation methodologies.

### 4.3. Strengths and Weaknesses

The primary strength of this study lies in its novelty as the first dedicated quantitative synthesis of the current evidence landscape. In contrast to previous reviews that have addressed the role of EVs in gynecological malignancies in a broad or qualitative manner [11], we provide a targeted meta-analysis specifically for cervical cancer, establishing a robust pooled AUC of 0.87. Furthermore, our strict adherence to PRISMA guidelines ensures a systematic and transparent appraisal of the literature. This methodological rigour not only minimizes selection bias but also confers a high degree of statistical power to our conclusions, distinguishing these findings from earlier descriptive summaries.

The primary weakness of the review is the significant heterogeneity and high risk of bias identified across the EV cargo analysis of the included studies. Although a meta-analysis was performed on the overall diagnostic accuracy of extracellular vesicles, the extreme variability in the types of EV-associated molecules (miRNAs, lncRNAs, and proteins) analysed and the lack of standardised protocols limited the capacity to draw definitive conclusions about the specific cargoes. This methodological inconsistency is compounded by inherent technical challenges in the field. As discussed by Thakur et al., the lack of standardized isolation and purification methods leads to variability in vesicle yield and purity, affecting the reproducibility of results. Furthermore, the co-isolation of non-vesicular contaminants, such as lipoproteins and protein aggregates, alongside difficulties in normalizing data from complex clinical biofluids, remains a critical hurdle for the clinical translation of EV biomarkers [45]. These factors ultimately affect the generalisability of results, requiring a cautious interpretation of the findings.

### 4.4. Implications for Practice and Future Research

The high diagnostic accuracy of extracellular vesicle biomarkers suggests their potential for future use in clinical practice as a non-invasive screening or diagnostic tool for cervical cancer, which could improve early detection rates and patient outcomes. However, the studies analyzed herein relied predominantly on standard characterisation techniques, such as Western blotting, transmission electron microscopy (TEM), and nanoparticle tracking analysis (NTA). While fundamental, these bulk methods often lack the sensitivity to capture stochastic heterogeneity or detect trace-level cargoes in complex clinical matrices. To bridge the gap towards clinical translation, future workflows must adopt a rigorous two-step paradigm: standardized isolation followed by high-resolution profiling. First, adherence to MISEV guidelines is imperative to ensure the reproducible enrichment of vesicles using established methods like ultracentrifugation or size exclusion chromatography [7].

Subsequently, the integration of advanced biophysical and nanoplasmonic platforms is required for detailed downstream characterisation. Atomic Force Microscopy (AFM) offers a distinct advantage in post-isolation assessment, enabling the nanomechanical analysis of vesicle size, morphology, and stiffness, thereby confirming the structural integrity of the isolates [46]. Complementarily, optical techniques such as Localized Surface Plasmon Resonance (LSPR) and Surface-Enhanced Raman Scattering (SERS) provide unparalleled sensitivity for molecular interrogation. Once vesicles are captured on sensor surfaces, LSPR facilitates label-free quantification, while SERS allows for the ‘molecular fingerprinting’ of specific cargoes, including HPV E6/E7 oncoproteins, at the single-vesicle level [47,48].

For future research, validating this integrated workflow of robust isolation and high-sensitivity detection in large-scale, prospective multicentric cohorts represents the requisite next step to confirm the diagnostic and prognostic utility of extracellular vesicles in routine oncological practice.

## 5. Conclusions

In conclusion, this systematic review and meta-analysis provide robust evidence for the diagnostic potential of extracellular vesicles as biomarkers for cervical cancer. While their promise as prognostic indicators is not yet fully established, the findings support a crucial shift towards more standardised methodologies to unlock the full clinical utility of these non-invasive biomarkers.

## Figures and Tables

**Table 1:** Inclusion and exclusion criteria based on PECO.

Rule	Inclusion Criteria	Exclusion Criteria
Study Type	Original research or replication studies published in peer-reviewed journals. No limitations imposed regarding publication date.	In vitro or animal studies without human data, case reports, review articles, editorials and per-spectives.
Participants	Non pregnant with HPV related cervical cancer. Older than 18 years. Without other invasive diseases. Non hospitalised. Studies involving all stages of cervical cancer. Studies involving CIN and/or healthy controls for comparison.	Under 18 years of age. Pregnant. Other invasive diseases. Only Animal models. Only Cell lines.
Exposure	Blood extracellular vesicles (EVs) Various types of EVs (e.g., exosomes, microvesicles, apoptotic bodies). EV-associated biomarkers (e.g., miRNA, nucleic acids, HPV DNA).	Without blood EVs. Examination of only cellular components without EV isolation. Documentation of EVs in cervical cancer, but without assessment of their biomarker potential.
Comparators	Healthy women. Cervical intraepitelial neoplasia. EV biomarkers across different stages of cervical cancer.	Absence of a clear comparison group or baseline. Comparison of only different EV isolation methods without clinical relevance.
Outcomes	Blood biomarker (EVs: miRNA, mRNA, lncRNAs HPVDNA or HPVRNA, ctDNA). Diagnostic biomarker (e.g., screening and/or early detection). Prognosis biomarker.	Other fluids: urine or cervicovaginal fluid. Absence of clear reporting of diagnostic or prognostic metrics. Focusing solely on therapeutic applications of EVs without biomarker assessment. Only qualitative outcomes without quantitative data.

**Table 2:** Characteristics of studies included in the study—outcome: Diagnostic.

Study (Author/ Year)	Country	Study Type	Sample Type	EVs Isolation Method	EVs Characterization Method (EVs Parameters)	EVs quantification Method	Type of Biomarker	Techniques for Biomarker Identification and Quantification	Specific Biomarker	Expression Level	Cases (N)	Healthy Controls (N)	CIN Controls (N)	Sensitivity (%)	Specificity (%)	AUC (IC 95%)	OR (IC 95%)	*p* Value	Included in the Meta-Analyses
Ding, Xian-zhen et al., 2020 [21]	China	Prospective cohort study	Serum	Total Exosome Isolation Kit (Invitrogen, CA, USA)	Western Blot (CD63, CD9, GM130)	Not performed	lncRNA	RNA extraction: miRNeasy Micro Kit (QIAGEN, Hilden, DE) RNA quality: Agilent 2100 Bioanalyzer RNA analysis: RT-qPCR (Taqman probes, Thermo Waltham, MA, USA)	lncRNA DLX6-AS1	Upregulated (significantly higher)	114	110	60	78.1% (CC vs. Healthy); 75.4% (CC vs. CIN)	88.2% (CC vs. Healthy); 71.8% (CC vs. CIN)	0.892 (95% CI: 0.844–0.929) for CC vs. Healthy; 0.831 (95% CI: 0.775–0.877) for CC vs. CIN	OS: Univariate analysis: HR = 3.54 (95% CI: 1.852–6.422, *p* = 0.008); Multivariate analysis: HR = 3.38 (95% CI: 1.742–6.178, *p* = 0.009)	Univariated: *p*: 0.008; multivariated: *p*: 0.009	yes
Guo Yaqiong 2020 [22]	China	Retrospective cross-sectional study	Serum	exoRNeasy Serum/ Plasma Midi Kit (QIAGEN, Hilden, DE)	Western Blot (CD9, CD63, GAPDH)	Not performed	lncRNA	RNA extraction: miRNeasy Kit	lncRNA-EXOC7	Higher level	101 (52 pre-operative/newly diagnosed cases, 30 post-operative cases, and 19 recurrence cases)	50	Not included	Not provided	Not provided	0.9388 for serum and 0.8982 for exosomal lncRNA-EXOC7	Not provided	0.041 for FIGO stage correlation with serum expression of lncRNA-EXOC7	yes
Cho O. et al., 2021 [23]	South Korea	Retrospective cohort study with a nested case-control element	Plasma	Exo2D (ExosomePlus, Seoul, Republic of Korea).	Not provided	Not performed	miRNA, mRNA, snoRNA	RNA extraction: RNeasy Serum/Plasma Kit (Qiagen)RNA quantification: Quant-IT Ribogreen (Invitrogen Carlsbad, CA, USA) RNA quality: Agilent 2100 Bioanalyzer RNA analysis: small RNA sequencing (Ilumina, San Diego, CA, USA)	13 miRNAs; 43 piRNAs; 28 lncRNAs, and 67 mRNAs →15 selected RNAs: miRNA: miR-142-3p; mRNAs: CXCL5, KIF2A, RGS18, ARL6IP5, and DAPP1; lncRNA: LINC00989 (via targeting RGS18); snoRNAs: SNORD17, SCARNA12, SNORA6, SNORA12, SCRNA1, SNORD97, SNORD62, and SNORD38A	Upregulated: RGS18. Downregulated: SNORA12; SNORD97	30	12	Not included	RGS18 + SNORA12 + SNORD97: 96.7%; RGS18: 96.7%; SNORA12 + SNORD97: 73.3%.	RGS18 + SNORA12 + SNORD97: 100%; RGS18: 91.7%; SNORA12 + SNORD97: 100%.	RGS18 + SNORA12 + SNORD97: 0.992; RGS18: 0.964; SNORA12 + SNORD97: 0.883	Not provided	*p* < 0.01	yes
Li et al., 2024 [24]	China	Retrospective cross-sectional study	Plasma	Ultracentrifugation	- Transmission electron microscopy (TEM) - Nanoparticle tracking analysis (NTA): qNano (Izon Science Ltd., New Zealand) - Western blot (CD9, GM130 and TSG 101)	Not performed	tRNA	RNA extraction: Trizol RNA analysis: RT-qPCR	tRF-Phe-GAA-001; tRF-Gly-GCC-037	Downregulated	140 (plasma)	140 (plasma)	Not included	For plasma exosomal tRF-Phe-GAA-001: 72.1%; tRF-Gly-GCC-037: 83.6%; Combined: 92.9%; For early stage: tRF-Phe-GAA-001: 80.2%% tRF-Gly-GCC-037: 81.5%	For plasma exosomal tRF-Phe-GAA-001: 85.7%; tRF-Gly-GCC-037: 69.3%; Combined: 83.6%; For early stage: tRF-Phe-GAA-001: 81.4% tRF-Gly-GCC-037: 69.3%	For plasma exosomal tRF-Phe-GAA-001: 0.8682; tRF-Gly-GCC-037: 0.8283; Combined: 0.9337 For early-stage CC plasma samples: tRF-Phe-GAA-001: 0.8963; tRF-Gly-GCC-037: 0.8103; Combined: 0.9432	Not provided	*p* < 0.0001 for both.	yes
Lv et al., 2021 [25]	China	Cross-sectional	Plasma	ExoEasy Maxi kit (Qiagen)	- Transmission electron microscopy (TEM) - Nanoparticle Tracking Analysis (NTA): ZetaView PMX	Not performed	miRNA	RNA extraction: *Discovery cohort:* miRNeasy Serum/Plasma Kit (Qiagen Hilden, DE, USA) *Validation cohort:* miRNeasy kit RNA analysis: *Discovery cohort:* miRNA sequencing (Illumina; San Diego, CA, USA); *Validation cohort:* RT-qPCR (syber green, giagen)	miR-125a-5p	Downregulated	*Discovery cohort:* 6 (cervical cancer) *Validation cohort:* 38 (cervical cancer)	*Discovery cohort:* 6 *Validation cohort:* 22	Not included	59.1% (at a cut-off value of 2.537 for miR-125a-5p)	84.2% (at a cut-off value of 2.537 for miR-125a-5p)	0.7129 (95% CI, 0.561–0.865)	Not provided	*p* < 0.001 for initial miRNA differential expression and *p* < 0.001 for difference in miR-125a-5p levels between cases and controls.	yes
Ma et al., 2019 [26]	China	Retrospective cross-sectional study	Plasma	ExoQuick exosome precipitation solution (System Biosciences, Palo Alto, CA, USA)	Not performed	Not performed	miRNA	RNA extraction: MirVana Paris Kit (Ambion, Austin, TX, USA)/Trizol RNA quantification: Quant-IT Ribogreen (Invitrogen, Carlsbad, CA, USA) RNA quality: Agilent 2100 Bioanalyzer RNA analysis: RT-qPCR (SYBR Green, TaKaRa, Kusatsu, Japan)	miR-146a-5p, miR-151a-3p, miR-2110, miR-21-5p (a four-miRNA panel). The expression of miR-21-5p was also increased in plasma exosomes of CC patients compared with those in NCs, albeit not reaching statistical significance.	Upregulated	24 (cervical cancer samples) All samples used for exosome analysis belonged to validation set.	24. All samples used for exosome analysis belonged to validation set.	Not included	Not provided	Not provided	Not provided for exossome samples	Not provided	miR-146a-5p: *p*: 0.041; miR-151a-3p *p*: 0.08; miR-2110: p.032; miR-21-5p: *p*: 0.079	no
Zheng et al., 2019 [27]	China	Retrospective cohort study with a nested case-control element	Plasma	ExoQuick Exosome Precipitation Solution (SBI Cat #:100356EXOQ20A-1, Mountain View, CA, USA) mixture with RNase A	Not provided	Not performed	miRNA	RNA extraction: *Discovery cohort:* miRNeasy Micro Kit *Validation cohort:* miRNeasy Micro Kit RNA quantification/quality: *Discovery cohort:* Agilent 2100 Bioanalyzer-Santa Clara, CA, USA; Qubit *Validation cohort:* Agilent 2100 Bioanalyzer; Qubit RNA analysis: *Discovery cohort:* miRNA sequencing (Ilumina, San Diego, CA, USA) *Validation cohort:* ddPCR	Eight miRNAs (let-7a-3p, let-7d-3p, miR-30d-5p, miR-144-5p, miR-182-5p, miR-183-5p, miR-215-5p, and miR-4443). Best predictors: let-7d-3p, miR-30d-5p	Down-regulated in CIN II+ compared to CIN I	*Discovery cohort:* 34 *Validation cohort:* 63 CC	*Discovery cohort:* 23 *Validation cohort: 50*	*Discovery cohort:* 5 CIN I, 59 CIN II-III	Not provided	Not provided	0.922 (for miRNA panel)	Not provided	Not provided	yes
Molika et al., 2023 [28]	Thailand	Retrospective cohort study with a nested case-control element	Serum	Differential ultracentrifugation (dUC) combined with size-exclusion chromatography (SEC)	Transmission electron microscopy (TEM) and Western blot (CD9, CD63, CD81, Cytocrome C)	Not performed	Protein	LC-MS/MS (Liquid Chromatography with tandem Mass Spectrometry)	ERI3, COX5A, SGSM3 (identified as uniquely expressed proteins in sEVs from CC patients; validation pending)	Up regulated (high level at CC)	90	30	Not included	Not provided	Not provided	Not provided	Not provided	several	no
Ao, K. et al., 2024 [29]	China	Cross-sectional	Plasma	*Discovery cohort:* ExoQuick test *Validation cohort:* Ultracentrifugation (UC)	Transmission electron microscopy (TEM) (morphology, size), Nanoparticle Tracking Analysis (NTA) (size)	Not performed	Protein	*Discovery cohort:* Label-free Quantitative LC/MS Proteomic analysis *Validation cohort:* Enzyme-linked immunosorbent assay (ELISA)	Mortalin (HSPA9)	Up regulated (high level at CC)	19 (prior to stage IIa)	15 (individuals with cervicitis as normal controls)	15 (patients with precancerous lesions—HSIL or CINII/III)	Not provided				*p* < 0.01	no

**Table 3:** Characteristics of studies included in the study: outcome: Prognostic.

Study (Author/Year)	Country	Study Type	Sample Type	EVs Isolation Method	EVs Characterization Method	EVs Quantification Method	Type of Biomarker	Techniques for Biomarker Identification and Quantification	Specific Biomarker	Expression Level	Cases (N)	Controls (N)	Sensitivity (%)	Specificity (%)	AUC (IC 95%)	OR (IC 95%)	*p* Value	Included in the Meta-Analyses
Guo, Yaqiong 2020 [22]	China	Retrospective cross-sectional study	Serum	exoRNeasy Serum/Plasma Midi Kit (QIAGEN, Hilden, Germany)	Western Blot (CD9, CD63, GAPDH)	Not performed	lncRNA	RNA extraction: miRNeasy Kit	lncRNA-EXOC7	Higher level	101 (52 pre-operative/newly diagnosed cases, 30 post-operative cases, and 19 recurrence cases)	50	Not provided	Not provided	0.9333	Not provided	Exosomal level of lncRNA-EXOC7 was correlated with the FIGO stage (*p* = 0.010)	no
Cho O. et al., 2021 [31]	South Korea	Prospective cohort study	Plasma	Exo2D (ExosomePlus, Seoul, Republic of Korea).	Not provided	Not performed	miRNA	RNA extraction: RNeasy Serum/Plasma Kit (Qiagen, Hilden, Germany) RNA quantification: Quant-IT Ribogreen (Invitrogen, Carlsbad, CA, USA) RNA quality: Agilent 2100 Bioanalyzer, Santa Clara, CA, USA. RNA analysis: small RNA sequencing (Ilumina, San Diego, CA, USA)	miR-1228-5p, miR-146a-3p, miR-33a-5p, miR-3200-3p, miR-501-3p, and miR-6815-5p	Upregulated	28 (cases: two weeks after the initiation of CCRT)	Before and after CCRT	Not provided	Not provided	Not provided	Not provided	Not provided	no
Molika, Piyatida et al. 2024 [32]	Thailand	Prospective Cohort Study (with cross-sectional elements)	Serum	Differential ultracentrifugation	- Transmission electron microscopy (TEM) - Western blot (CD9, CD63, Alix, albumin, and cytochrome C)	Not performed	lncRNA	RNA extraction: ExoRNeasy Kit (Qiagen, Hilden, Germany) RNA quantification: Agilent 2100 Bioanalyzer RNA quality: Agilent 2100 Bioanalyzer Santa Clara, CA, USA RNA analysis: small RNA sequencing (Ilumina, San Diego, CA, USA)	LINC00941, LINC00482, LINC01116, LINC02274, LINC01489, LINC01910, LINC02454, DSG2-AS1, HCG15, TCEAL3-AS1, LINC01960, LINC01398, LINC02251, RNASEH1-AS1, and LINC01271	Upregulated	60 (Cervical cancer patients, staged I–III)	27 healthy	Not provided	Not provided	Not provided	Not provided	*p* < 0.05	no
Someya et al., 2023 [30]	Japan	Prospective Cohort Study (with cross-sectional elements)	Plasma	exoRNeasy Serum/Plasma Kit (QIAGEN, Hilden, Germany)	Not performed	Not performed	miRNA	RNA quality: Agilent 2100 Bioanalyzer, Santa Clara, CA, USA RNA analysis: mir-RNA sequencing (Ilumina, San Diego, CA, USA)	Nine-miRNA signature (miR-148a-5p, miR-1915-3p, miR-3960, miR-183-5p, miR-196b-5p, miR-200c-3p, miR-182-5p, miR-374a-5p, miR-431-5p)	Down expression at recurrence: miR-148a-5p; miR-1915-3p; miR-3960; UP expression: miR-183-5p; miR-183-5p; miR-200c-3p; miR-182-5p; miR-374a-5p miR-431-5p	45	Recurrence (19: 15 deaths) vs. non recurrence (26)	Not provided	Not provided	miR-148a-5p: 0.713 miR-1915-3p: 0.682 miR-3960: 0.806;miR-183-5p: 0.680; miR-183-5p 0.686; miR-200c-3p 0.721; miR-182-5p 0.709; miR-374a-5p 0.729; miR-431-5p: 0.729	miR-148a-5p: 4.7 miR-1915-3p: 4.1 miR-3960: 22.4;miR-183-5p: 3.8; miR-183-5p: 5.9; miR-200c-3p: 11.9; miR-182-5p 5.9; miR-374a-5p 5.9; miR-431-5p: 6.3	miR-148a-5p: 0.007 miR-1915-3p: 0.031 miR-3960: >0.001; miR-183-5p: 0.027; miR-183-5p: 0.028; miR-200c-3p: 0.006; miR-182-5p: 0.007; miR-374a-5p: 0.003; miR-431-5p: 0.003	no

**Table 4:** Patient Characteristics: Outcome: Diagnosis.

Study/ Cases (N)/ Histology	Age ^a^ CC (Years)	FIGO Staging (N)	Healthy Controls (N)	CIN Controls (N)	HPV Infection Cases	EV Biomarker	Main Findings
[21] N: 114 (Histology not provided)	<50:56 ≥50:58	I–II: 79 III–IV: 35	110	60	Negative: 17 Positive: 97	lncRNA DLX6-AS1	Serum exosomal lncRNA DLX6-AS1 levels were significantly higher in CC patients than in CIN and healthy controls (both *p* < 0.001); Higher serum exosomal lncRNA DLX6-AS1 levels were observed in CIN patients compared with controls (*p* = 0.011).
[22] ^b^ N: 101 (Histology not provided)	<50:51 ≥50:50	IB–IIA: 38 IIB–IIIA: 27 IIIB–IV: 36	50	Not included	Not provided	lncRNA-EXOC7 (serum and exosomal)	Newly diagnosed CC cases presented a 3.18-fold higher serum level of lncRNA-EXOC7 and a 2.93-fold higher exosomal level of lncRNA-EXOC7 than healthy subjects. ROC curve analysis showed that the AUCs of serum and exosomal lncRNA-EXOC7 in distinguishing CC patients from healthy controls were 0.9338 and 0.8982, respectively. Exosomal level was also elevated in recurrent cases (*p* < 0.0001, AUC = 0.9123).
[23] N: 30: SCC: 22; AC: 4; ACC 2; UC: 2	49.2 ± 11.6		12	Not included	Not provided	miRNA: miR-142-3p; mRNAs: CXCL5, KIF2A, RGS18, APL6IP5, DAPP1; snoRNAs: SNORD17, SCARNA12, SNORA6, SNORA12, SCRNA1, SNORD97, SNORD62, SNORD38A	SNORA12: the most relative to ALC2 among other snoRNAs, which showed that snoRNA was more suitable for cancer diagnose than the other three RNA classes (e.g., snRNA, tRNA, and yRNA) because it had an additional four RNAs that were differentially expressed in the cancer group.
[24] N: 140 SCC: 96; AC: 44	<55:72 ≥55:68	I–IIA:81 IIB–IV: 59	140	Not included	Positive: 129 Negative: 11	tRF-Phe-GAA-001; tRF-Gly-GCC-037	Significant decrease in the levels of exosomal tRF-Phe-GAA-001 and tRF-Gly-GCC-037 compared to healthy controls (*p* < 0.0001 for both). combination, the AUC rose to 0.9337, with a sensitivity of 92.9% and specificity of 83.6%.
[25] N: 38 (SCC:28; AC: 10)	<50:6 ≥50:32	IA–IIA: 29 IIB–IVB: 9 Missing: 8	22	Not included	Positive: 30 Negative: 8	miR-125a-5p	Level of plasma exosomal miR-125a-5p was a potential marker for differentiating between non-cervical and cervical cancer, with an ROC area under the curve value of 0.7129 [95% confidence interval (CI), 0.561–0.865]
[26] N: 97 (combined phase) (SCC: 76; AC: 21)	≥60:12 <60:85	I: 74 II: 23	87	Not included	Not provided	miR-146a-5p, miR-151a-3p, miR-2110, miR-21-5p	The expression of miR-21-5p was increased in plasma exosomes of CC patients compared with those in NCs, albeit not reaching statistical significance.
[27] N: 34 Cases defined as CIN II + (59 CINII-III + 34 CC: SCC: 21 and AC:13)	50 ± 24	Not provided	23	5	Not provided	Eight miRNAs (let-7a-3p, let-7d-3p, miR-30d-5p, miR-144-5p, miR-182-5p, miR-183-5p, miR-215-5p, and miR-4443). Best predictors (plasma and tissue): let-7d-3p and miR-30d-5p	AUC for miRNA panel, discriminate CIN II+ from Healthy/CIN I: 0.922. Individual AUC DEmiRs: let-7a-3p: 0.811; let-7d-3d: 0.888; mir-30d-5p: 0.915; mir-215-5p: 0.817; miR-144-5p: 0.882; miR-182-5p: 0.773; miR-183-5p: 0.814; miR-443: 0.817.
[28] N: 90 (Histology not provided)	30–65	Not provided	30	Not included	Not provided	ERI3, COX5A, SGSM3	ERI3, COX5A, and SGSM3 are uniquely expressed proteins in EVs derived from patients with CC.
[29] N: 19 SCC	43.6 (24–70)	IA1:5 IB1:4 IIA: 3 IIB: 1 Missing: 6	15	15: CINII: 4 CINIII: 4	HPV 16: 12 HPV 18: 1 HPV 31: 2 HPV 58: 2 Negative: 2	Mortalin (or HSPA9)	The CC group boasted a higher content of mortalin in comparison to the HSIL or Control (*p* < 0.01). Promising target for clinical interventions as well as an early warning indicator.

^a^ Based on study data; ^b^ Also included at prognosis outcome characterization on samples only at Table 2; AC: Adenocarcinoma; AUC: Area Under the Curve; DEmiRs: differently expressed miRs; NC: normal controls. SCC: Squamous Cell carcinoma.

**Table 5:** Patient Characteristics: Outcome: Prognosis.

Study/Cases (N)/Histology	Age CC (Years)	FIGO Staging (N)	Controls (N)	EV Biomarker	Main Findings
[30] N: 45 SCC: 44 AC: 1	44–79 (56)	I: 2 IIa + IIb: 9 IIIa + IIIb: 17 IVa + IVb: 17	Recurrence-free (26) and recurrence (19)	Lower expression recurrence: Higher expression: miR-183-5p, miR-200c-3p, miR-182-5p, miR-374a-5p, miR-431-5p	miRNA-based risk score, along with tumor size and infiltrating FOXP3+ T cells, were significant factors in predicting disease-specific survival. (*p* < 0.001).
[31] N:28 SCC: 22 AC: 4 ACC: 1; UC 1	50.0 (42.5; 56.0)	IB: 4 IIB: 1 IIIC1: 13 IIIC2: 6 IVA: 1 IVB: 3	Before and after two weeks of treatment	miR-1228-5p, miR-146a-3p, miR-33a-5p, miR-3200-3p, miR-501-3p, and miR-6815-5p	log2FC of miRNAs contained within plasma exosomes are associated with early progression and metastasis in patients with cervical cancer. miR-16-1-3p (or 15a-3p) may be a potential upstream regulator important for the suppression of metastasis by modulation of the tumor microenvironment.
[32] N: 60 Not provided	Not provided		27 healthy controls	LINC00941, LINC01910, LINC02454	The EV lncRNAs upregulated in stage III patients compared with those in HC are involved in multiple biological processes, including HPV infection, MAPK signaling pathway, and focal adhesion. High expression of LINC00941, LINC01910, LINC02454, and DSG2-AS1 were associated with poor OS of patients with CC.

AC: Adenocarcinoma; ACC: Adenosquamous cell carcinoma: OS: Overall Survival; RT: radiotherapy; SCC: Squamous Cell carcinoma; UC: Unclassified carcinoma; HC: healthy control.

**Table 6:** GRADE assessment for the certainty of evidence.

Outcomes ^a^	Study Design	Risk of Bias in Individual Studies	Publication Bias	Inconsistency	Indirectness	Imprecision	Confidence in Evidence	Recommendation
Diagnosis and/or Prognosis	12 observational studies.	High ^b^	No bias detected ^c^	High ^d^	High ^e^	High ^f^	⊕ Very low.	No recommendation can be provided based on existing data.

^a^ Outcomes were grouped as their assessments were not different. ^b^ Detailed assessments in Table 5. ^c^ Assessed through extended Egger’s test, *p*: 0.436. ^d^ Assessed through *I^2^* = 82.9% but also considering qualitative analysis from studies not included in meta-analysis. Because the outcomes are nominal variables, high heterogeneity was expected. Heterogeneity also likely emerged from very distinct study designs. ^e^ Several issues identified: Sample heterogeneity: Mixed histological subtypes (e.g., squamous cell carcinoma vs. adenocarcinoma) and stages (CIN I–III vs. early-stage (I–II) to late-stage (III–IV)). HPV status reporting gaps: Critical for cervical cancer biomarker validity, as HPV-driven tumors exhibit distinct exosomal signatures. Methodological disparities: Ultracentrifugation isolates larger exosomes (>100 nm), while kit-based methods (e.g., ExoQuick™) recover smaller vesicles with protein aggregates. Missing reference standards: No consensus on “normal” exosome levels in plasma for healthy vs. diseased states. ^f^ While some imprecision is expected due to the variables included, additionally, the heterogenous and small sample size, generate uncertainty about magnitude of effect.

**Table 7:** Risk assessment.

	Risk of Bias				Applicability Concerns		
Study (Author/Year)	Patient Selection	Index Test	Reference Standard	Flow and Timing	Patient Selection	Index Test	Reference Standard
Ao, K. et al., 2024 [29]	☹	☺	☺	☺	☹	?	☺
Cho O.et al., 2021 [23]	☹	☹	☺	☺	☹	?	☺
Ding.Xian-zhen et al., 2020 [21]	☹	☺	☺	☺	☹	?	☺
Guo Ya-qiong, 2020 [22]	☹	?	☺	☺	☹	?	☺
Li et al., 2024 [24]	☹	☺	☺	☺	☹	?	☺
Lv et al., 2021 [25]	☹	☺	☺	☺	☹	?	☺
Ma et al., 2019 [26]	☹	☺	☺	☺	☹	?	☺
Molika et al., 2023 [28]	☹	☺	☺	☺	☹	?	☺
Zheng et al., 2019 [27]	☹	☺	☺	☺	☹	?	☺
Cho O. et al., 2021 [31]	☹	?	☺	☺	☹	?	☺
Molika, Piyatida et al., 2024 [32]	☹	☺	☺	☺	☹	?	☺
Someya et al., 2023, [30]	☹	?	☺	☺	☹	?	☺

☺ Low Risk, ☹ High Risk, ? Unclear Risk.

## Data Availability

No new data were created or analysed in this study. Data sharing is not applicable to this article as all data were obtained from previously published studies, which are cited within the manuscript.

## Abbreviations

The following abbreviations are used in this manuscript:

CC	cervical cancer
EVs	Extracellular Vesicles
AUC	Area Under the Curve
miRNAs	microRNAs
lncRNAs	long non-coding RNAs
ctDNA	circulating tumour DNA
